# Evaluating Retinal and Choroidal Perfusion Changes after Isometric and Dynamic Activity Using Optical Coherence Tomography Angiography

**DOI:** 10.3390/diagnostics11050808

**Published:** 2021-04-29

**Authors:** Max Philipp Brinkmann, Nikolas Xavier Kibele, Michelle Prasuhn, Vinodh Kakkassery, Mario Damiano Toro, Mahdy Ranjbar, Salvatore Grisanti, Matthias Becker, Felix Rommel

**Affiliations:** 1 Department of Ophthalmology, Stadtspital Waid und Triemli Zürich, 8063 Zürich, Switzerland; max.brinkmann@gmx.de (M.P.B.); n.x.kibele@outlook.com (N.X.K.); matthias.becker@triemli.zuerich.ch (M.B.); 2 Department of Ophthalmology, Klinikum Klagenfurt, A-9020 Klagenfurt, Austria; 3 Department of Ophthalmology, University of Lübeck, 23538 Lübeck, Germany; michelle.prasuhn@uksh.de (M.P.); vinodh.kakkassery@uksh.de (V.K.); eye.research101@gmail.com (M.R.); salvatore.grisanti@uksh.de (S.G.); 4 Department of Ophthalmology, University Hospital Zurich, University of Zurich, 8091 Zurich, Switzerland; 5 Faculty of Medical Sciences, Collegium Medicum, Cardinal Stefan Wyszyński University, 01815 Warsaw, Poland; 6 Department of Ophthalmology, University of Heidelberg, 69120 Heidelberg, Germany

**Keywords:** isometric activity, dynamic activity, optical coherence tomography angiography, retinal perfusion, choroidal perfusion, sports medicine

## Abstract

Optical coherence tomography angiography (OCTA) is a non-invasive tool for imaging and quantifying the retinal and choroidal perfusion state in vivo. This study aimed to evaluate the acute effects of isometric and dynamic exercise on retinal and choroidal sublayer perfusion using OCTA. A pilot study was conducted on young, healthy participants, each of whom performed a specific isometric exercise on the first day and a dynamic exercise the day after. At baseline and immediately after the exercise, heart rate (HR), mean arterial pressure (MAP), superficial capillary plexus perfusion (SCPP), deep capillary plexus perfusion (DCPP), choriocapillaris perfusion (CCP), Sattlers’s layer perfusion (SLP), and Haller’s layer perfusion (HLP) were recorded. A total of 34 eyes of 34 subjects with a mean age of 32.35 ± 7.87 years were included. HR as well as MAP increased significantly after both types of exercise. Both SCPP and DCPP did not show any significant alteration due to isometric or dynamic exercise. After performing dynamic exercise, CCP, SLP, as well as HLP significantly increased. Changes in MAP correlated significantly with changes in HLP after the dynamic activity. OCTA-based analysis in healthy adults following physical activity demonstrated a constant retinal perfusion, supporting the theory of autoregulatory mechanisms. Dynamic exercise, as opposed to isometric activity, significantly changed choroidal perfusion. OCTA imaging may represent a novel and sensitive tool to expand the diagnostic spectrum in the field of sports medicine.

## 1. Introduction

During the last few years, optical coherence tomography angiography (OCTA) has contributed significantly to the diagnostic scope within ophthalmology by visualizing the retinal and choroidal vascular network in a non-invasive fashion [1,2,3,4]. Besides the advantage of a harmless examination without side effects, OCTA allows high-resolution three-dimensional mapping of the microvasculature within variable slabs [2,5]. Due to the potential of visualizing microcapillaries in vivo, OCTA has also impact on other medical disciplines, since many systemic disorders lead to early vascular involvement of the ocular vessels of the posterior pole [6,7,8,9]. Moreover, the effect of changes in the systemic circulation on retinal and choroidal perfusion metrics represents a field of increasing interest for OCTA research. It has been demonstrated that acute Valsalva maneuvers as well as long-lasting arterial hypertension significantly influence the retinal perfusion examined by OCTA [10,11].

So far, little research has been done in sports medicine regarding perfusion changes examined by OCTA. It is well known that regular physical activity has widespread health benefits such as decreased risk for coronary artery disease, diabetes mellitus, and hypertension [12,13]. Furthermore, higher levels of physical activity are associated with a lower prevalence of retinal microvascular abnormalities, and regular exercise can even reverse subclinical impairment of the retinal microvasculature [14,15]. Alnawaiseh et al. were the first to find significant changes of retinal perfusion by using OCTA after a short-term exercise, consisting of sit-ups, pushups, squats, lunges, and rope skipping [16]. However, by using a conventional training program, the authors did not distinguish between isometric and dynamic exercise. Furthermore, the retinal blood flow is mainly determined by autoregulatory mechanisms and local factors, while the choroidal circulation is controlled by autonomic innervation and directly influenced by systemic perfusion changes [17,18,19]. Therefore, the aim of this study was to evaluate the impact of changes in systemic circulation due to a specific isometric as well as a dynamic exercise on retinal and choroidal sublayer perfusion as measured by OCTA.

## 2. Materials and Methods

In this pilot study, 34 eyes of 34 healthy participants were included. Exclusion criteria were: (1) evidence or history of systemic disorders including cardiovascular diseases, diabetes mellitus, as well as neurological disorders; (2) evidence of systemic drug use; (3) history or evidence of ocular disease, ocular surgery, or local medication. Laterality was assigned by chance, leading to 15 included right eyes and 19 left eyes. All participants were enrolled and examined at the Triemli City Hospital Zurich between July 2019 and December 2019. The study was approved by the local Ethics Committee (vote number 2019-00724 on 17 June 2019) and followed the tenets of the Declaration of Helsinki. Before enrollment, all participants were informed about the study protocol, and informed consent was obtained individually. 

**Figure 1:**
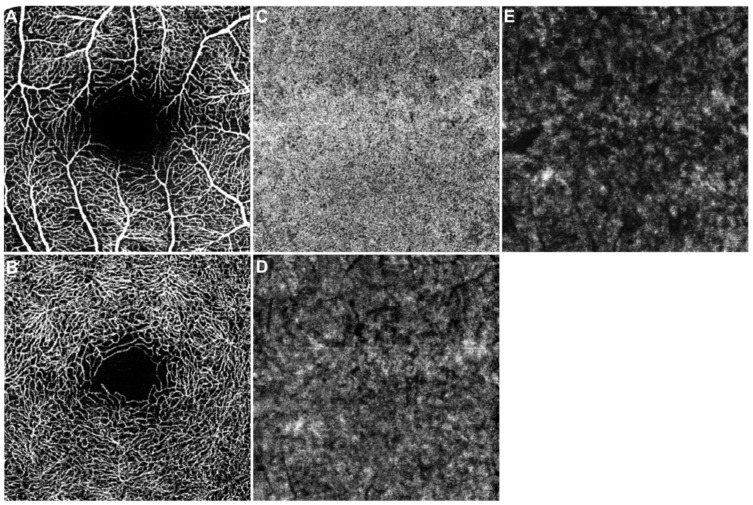
OCTA-imaging of the posterior pole in a healthy participant (15° × 15°). En face angiogram (raw data) of the superficial capillary plexus (**A**), deep capillary plexus (**B**), choriocapillaris (**C**), Sattler’s layer (**D**), and Haller’s layer (**E**).

After enrollment, all included participants underwent a complete ocular examination including refraction, best-corrected visual acuity (BCVA) in Snellen and slit-lamp biomicroscopy. Only eyes with maximum permissible spherical and cylindrical aberration of ±3 and ±1 diopters, respectively, and BCVA of at least 20/25 were included. Furthermore, the included study eye was examined by OCTA. The imaging was performed without prior pupil dilatation using the SPECTRALIS OCT Angiography Module (Heidelberg Engineering, Heidelberg, Germany) by a single, trained operator (N.X.K). The imaging session included a 15° × 15° (~4.4 × 4.4 mm) scan pattern of the posterior pole, centered on the fovea. To avoid bias due to physiological diurnal changes of the ocular perfusion, all examinations were carried out around noon [17,20,21]. Furthermore, only OCTA scans with good quality and without motion, segmentation, and projection artifacts were accepted in the data analysis [22]. After acquisition, OCTA images were automatically segmented according to the manufacturer’s default setting to create en face images of the retinal superficial capillary plexus (SCP) and the deep capillary plexus (DCP) (Figure 1A,B). The SCP extended from the internal limiting membrane to the inner plexiform layer (IPL), while the DCP was defined between the IPL and the outer plexiform layer. To create specific choroidal flow maps, the OCTA images were manually segmented following previous published protocols [23,24,25,26,27]. Briefly summarized, OCTA images were manually segmented to create 20 µm slabs of the choriocapillaris (CC), Sattler’s layer (SL), and Haller’s layer (HL) (Figure 1C–E). Each retinal and choroidal angiogram was subsequently exported into ImageJ (NIH, Version 1.53e, Bethesda, MA, USA) and binarized by the Otsu method to count the percentage of black and white pixels [28]. The perfusion of the SCP (SCPP), DCP (DCPP), and CC (CCP) was determined by recording the white pixels in relation to the overall image, while for perfusion of SL (SLP) and HL (HLP), the black pixels were taken into account, as published before [27,29].

After all baseline examinations were carried out, participants were asked to rest for 5 min. Systemic systolic and diastolic blood pressure (BP) as well as heart rate (HR) were measured in the left brachial artery in an upright sitting position. Participants with baseline BP above 140 mmHg systolic or 90 mmHg diastolic were excluded from the study as well. At least 1 h before the exercise, the individually maximal voluntary contraction (MVC) was measured with an isometric handgrip. The participants were asked to perform a 2 min isometric exercise bout at 30% MVC with an isometric handgrip, following a previous published protocol [30]. Immediately after the activity, BP, HR, as well as OCTA imaging were performed in the same manner. 

On the next day, the participants underwent another baseline examination including BP, HR, and OCTA imaging of the study eye after resting for 5 min. Afterwards, the participants were asked to perform a standardized dynamic activity, consisting of stair ascending and descending over 3 floors with 20 stairs on each floor. Again, immediately after finishing the exercise, examinations were repeated.

Microsoft Excel 2016 (Microsoft Corp., Redmond, WA, USA) was used for the management of the obtained data. Statistical analyses were performed using IBM SPSS 27.0 (IBM Corp., Armonk, NY, USA) and GraphPad PRISM 9.0 (GraphPad Software, Inc., San Diego, CA, USA). Power calculation was performed by using G*Power (Version 3.1, Düsseldorf, Germany), and a sample size of 30 was calculated to achieve a power of at least >95%. BCVA in decimal Snellen was converted to the logarithm of the minimum angle of resolution (logMAR). Mean arterial pressure (MAP) was calculated based on the formula 2/3 diastolic BP + 1/3 systolic BP. The Shapiro–Wilk test was used to check for normal distribution of the obtained data. As data were found to be distributed normally, quantitative data were summarized as mean ± standard deviation (SD), and qualitative variables as frequency and percentage. Functional parameters before and after exercise were compared using *t*-tests for paired data. Possible correlation between the difference in MAP, HR, and perfusion values due to isometric or dynamic activity was expressed as Pearson correlation coefficient. For all statistical tests, values of *p* < 0.05 were considered statistically significant.

## 3. Results

A total of 34 eyes of 34 healthy participants were included in this analysis. Demographic and clinical data are reported in Table 1. Four male (11.8%) and 30 female (88.2%) participants with a mean age of 32.35 (±7.87) years were included.

After the isometric activity, the mean MAP significantly increased from 109.5 (±11.8) to 117.3 (±18.7) mmHg (*p* = 0.007), and the HR increased from 71.2 (±11.3) to 79.6 (±23.5) bpm (*p* = 0.021). The perfusion values of both retinal plexus and choroidal sublayers did not change significantly due to the isometric activity (Table 2).

After performing dynamic exercise, the mean MAP significantly increased from 106.2 (±10.7) to 127.6 (±16.3) mmHg (*p* < 0.001), and the HR increased from 68.2 (±11.7) to 88.9 (±26.5) bpm (*p* < 0.001). The perfusion of the two retinal plexus did not change significantly after the activity. All choroidal sublayers showed an increased perfusion immediately after performing the dynamic exercise. While CCP increased from 40.88 (±4.0) to 42.23 (±4.03) % (*p* < 0.001), SLP (*p* < 0.001) and HLP (*p* < 0.001) increased from 75.32 (±7.1) to 76.93 (±7.45) % and from 87.99 (±3.86) to 89 (±4.07) %, respectively (Table 3).

Differences in MAP, HR, and perfusion metrics due to isometric and dynamic exercise were correlated in Table 4 and Table 5, respectively. After isometric activity, differences in MAP and HR (r = 0.497; *p* = 0.003), as well as SCPP and DCPP (r = 0.833; *p* < 0.001) and SLP and HLP (r = 0.8; *p* < 0.001) significantly correlated positively. Furthermore, after dynamic exercise, a significant positive correlation was observed between the difference in MAP and HLP (r = 0.413; *p* = 0.017).

## 4. Discussion

In this pilot OCTA-based study, we investigated retinal and choroidal sublayer perfusion alterations in healthy eyes immediately after performing an isometric and a dynamic exercise. To the best of our knowledge, this is the first study demonstrating statistically significant increases of perfusion in all choroidal sublayers after dynamic exercise, while an isometric exercise did not lead to ocular perfusion changes. Furthermore, the perfusion changes noted in HL directly correlated with changes in MAP after performing the dynamic activity.

Isometric exercise, in contrast to dynamic exercise, describes the activity of a muscle without any movement of the surrounding joints. By applying constant tension to the muscles, isometric exercise can be useful for muscle strengthening and stabilization [31,32]. Furthermore, it is well documented that both isometric and dynamic exercise elicit physiological responses such as increased HR, MAP, cardiac output, and sympathetic nerve activity [30,31]. However, the systemic arterial pressure response to both types of exercise seems to be different. Iellamo et al. indicated that exercise of a dynamic type induces greater MAP response than intensity-matched isometric exercise in healthy, young adults [33]. These published data are in line with our results, as the participants had a greater increase of MAP and HR after performing a dynamic activity. 

After performing isometric or dynamic exercises, retinal perfusion did not change significantly either in the SCP or in the DCP. Alnawaiseh et al. demonstrated a significant decrease of the central retinal flow density by using OCTA after a specific training program mainly consisting of dynamic exercises [16]. However, in contrast to our study protocol, the dynamic exercise in their study was more intensive, leading to a higher increase of HR and MAP. The authors explained the decrease of retinal perfusion by increased energy consumption and redistribution of blood supply as well as by blood being diverted to other organs during exercise. However, previous OCTA- and laser doppler velocimetry-based studies already demonstrated that changes in systemic perfusion pressure have only a negligible influence on retinal blood flow [17,34,35,36]. The absence of neuronal innervation in retinal vascular beds seems to be the responsible mechanism for the insensitivity to systemic changes in MAP within the retinal circulation. While histological studies have revealed a rich supply of autonomic vasoactive innervation for the choroidal vascular network, the nerves do not go any further into the retina [19]. Hence, retinal blood flow is mainly controlled by myogenic and local metabolic autoregulatory mechanisms. The results of the present study support the theory of a steady retinal blood flow due to autoregulation without influence by changes of systemic BP. 

Contrary to the retinal vessels, the choroid has a large amount of autonomic vasoactive innervation with low autoregulatory capability, making MAP the main influencing factor for choroidal perfusion [37]. We were able to confirm this theory by demonstrating a significant increase of perfusion in all choroidal sublayers after performing dynamic exercise with a significant increase of MAP from 106.2 to 127.6 mmHg (*p* < 0.001). Furthermore, perfusion changes of HL directly correlated with changes of MAP. However, we were only able to demonstrate an increased choroidal perfusion after performing dynamic exercise and not after isometric exercise. An explanatory approach could be the lower increase of BP after isometric activity compared to dynamic exercise. Furthermore, an important influencing factor of choroidal perfusion in addition to BP is intraocular pressure (IOP) [37]. Several studies were able to demonstrate a transient decrease of IOP in the acute postexercise period [38,39]. Furthermore, the lowering effect of IOP seems to depend on the intensity of the exercise [40]. In the present study, one could assume that isometric exercise led to a lower postexercise IOP drop than dynamic exercise. Therefore, the combination of a higher MAP increase and an IOP decrease after performing the dynamic exercise could explain the changes in choroidal sublayer perfusion. However, in addition to IOP, future studies should try to continuously monitor HR and MAP during exercise. This way, both types of exercise can be adjusted to increase HR and MAP by the same amount, so to increase the comparability of the ocular perfusion metrics.

The present study may be limited for several reasons. We only included young and healthy participants; therefore, the results cannot be transferred directly to patients with ocular pathologies or systemic diseases. Furthermore, possible gender-specific differences regarding ocular perfusion changes were not taken into account when recruiting participants, resulting in an unbalanced gender ratio. By not recording IOP before and after exercise, we may have missed out on an important potential factor with influence on choroidal perfusion. Participants were asked to perform the isometric exercise the day before performing the dynamic exercise. It cannot be ruled out that the baseline perfusion state on the second day was influenced by the isometric activity. Furthermore, potential influencing factors on OCTA analysis such as hematocrit, caffeine, and hormone status were not recorded [41,42]. In addition, perfusion values may differ from device to device, depending on hardware, segmentation, and software algorithms. The levels of segmentation should therefore be checked when comparing data between devices. Moreover, the understanding and interpretation of various signals in the choroidal vasculature on OCTA analysis are controversial and need further research. Finally, the relatively small sample size may represent a limiting factor leading to a mainly exploratory data analysis. Nevertheless, strong significant perfusion changes were found.

## 5. Conclusions

In conclusion, this pilot study demonstrated a significant increase of perfusion in all choroidal sublayers after performing a dynamic exercise, while retinal perfusion stayed steady. These findings support the theory of autoregulatory mechanisms and local metabolites controlling the retinal blood flow, whereas the choroidal blood flow mainly depends on systemic MAP. OCTA imaging may represent a novel and sensitive tool to expand the diagnostic spectrum in the field of sports medicine.

## Figures and Tables

**Table 1:** Demographic and clinical data of the enrolled participants.

Parameter	
*n* (subjects)	34
Age (years)	32.35 ± 7.87
Sex (M/F)	4/30 (11.8%/88.2%)
BCVA (logMAR)	−0.07 ± 0.05

F, female; M, male; BCVA, best-corrected visual acuity; SD, standard deviation; values are given as mean ± SD unless otherwise specified.

**Table 2:** Functional parameters before and after isometric activity.

Parameter	Before Activity Mean (± SD)	After Activity Mean (± SD)	Paired *t*-Test *p*-Value
MAP (mmHg)	109.5 (± 11.8)	117.3 (± 18.7)	0.007
HR (bpm)	71.2 (± 11.3)	79.6 (± 23.5)	0.021
SCPP (%)	31.47 (± 5.42)	31.70 (± 4.96)	0.638
DCPP (%)	27.91 (± 4.51)	28.42 (± 4.22)	0.256
CCP (%)	41.38 (± 2.98)	42.00 (± 3.49)	0.124
SLP (%)	76.24 (± 7.64)	75.93 (± 7.85)	0.360
HLP (%)	88.99 (± 4.98)	88.66 (± 5.12)	0.146

MAP, mean arterial pressure; HR, heart rate; SCPP, superficial capillary plexus perfusion; DCPP, deep capillary plexus perfusion; CCP, choriocapillaris perfusion; SLP, Sattler’s layer perfusion; HLP, Haller’s layer perfusion; SD, standard deviation.

**Table 3:** Functional parameters before and after dynamic activity.

Parameter	Before Activity Mean (± SD)	After Activity Mean (± SD)	Paired *t*-Test *p*-Value
MAP (mmHg)	106.2 (± 10.7)	127.6 (± 16.3)	<0.001
HR (bpm)	68.2 (± 11.7)	88.9 (± 26.5)	<0.001
SCPP (%)	32.47 (± 5.99)	33.26 (± 5.67)	0.344
DCPP (%)	29.19 (± 5.05)	29.59 (± 4.15)	0.627
CCP (%)	40.88 (± 4.00)	42.23 (± 4.03)	<0.001
SLP (%)	75.32 (± 7.10)	76.93 (± 7.45)	<0.001
HLP (%)	87.99 (± 3.86)	89.00 (± 4.07)	<0.001

MAP, mean arterial pressure; HR, heart rate; SCPP, superficial capillary plexus perfusion; DCPP, deep capillary plexus perfusion; CCP, choriocapillaris perfusion; SLP, Sattler’s layer perfusion; HLP, Haller’s layer perfusion; SD, standard deviation.

**Table 4:** Correlation analysis of changes in arterial pressure, heart rate, and ocular perfusion values due to isometric activity.

Parameter		MAP	HR	SCPP	DCPP	CCP	SLP	HLP
MAP	CC *p*	1	0.497 0.003	0.005 0.977	0.017 0.923	−0.059 0.739	0.076 0.670	0.031 0.860
HR	CC *p*	0.497 0.003	1	−0.148 0.402	0.050 0.777	0.064 0.718	−0.165 0.353	−0.071 0.690
SCPP	CC *p*	0.005 0.977	−0.148 0.402	1	0.833 <0.001	0.272 0.120	−0.050 0.777	−0.007 0.967
DCPP	CC *p*	0.017 0.977	0.050 0.777	0.833 <0.001	1	0.430 0.081	−0.278 0.112	−0.043 0.809
CCP	CC *p*	−0.059 0.739	0.064 0.718	0.272 0.120	0.430 0.081	1	−0.452 0.070	−0.195 0.270
SLP	CC *p*	0.076 0.670	−0.165 0.353	−0.050 0.777	−0.278 0.112	−0.452 0.070	1	0.800 <0.001
HLP	CC *p*	0.031 0.860	−0.071 0.690	−0.007 0.967	−0.043 0.809	−0.195 0.270	0.800 <0.001	1

MAP, mean arterial pressure; HR, heart rate; SCPP, superficial capillary plexus perfusion; DCPP, deep capillary plexus perfusion; CCP, choriocapillaris perfusion; SLP, Sattler’s layer perfusion; HLP, Haller’s layer perfusion; CC, correlation coefficient; *p*, *p*-value.

**Table 5:** Correlation analysis of changes in arterial pressure, heart rate, and ocular perfusion values due to dynamic activity.

Parameter		MAP	HR	SCPP	DCPP	CCP	SLP	HLP
MAP	CC *p*	1	0.132 0.456	−0.058 0.745	−0.189 0.285	−0.031 0.886	0.185 0.303	0.413 0.017
HR	CC *p*	0.132 0.456	1	−0.051 0.776	−0.112 0.530	0.038 0.836	−0.291 0.101	−0.161 0.372
SCPP	CC *p*	−0.058 0.745	−0.051 0.776	1	0.877 <0.001	0.196 0.283	0.502 0.003	0.279 0.116
DCPP	CC *p*	−0.189 0.285	−0.112 0.530	0.877 <0.001	1	0.096 0.600	0.458 0.007	0.221 0.216
CCP	CC *p*	−0.031 0.866	0.038 0.836	0.196 0.283	0.096 0.600	1	0.336 0.060	−0.125 0.496
SLP	CC *p*	0.185 0.303	−0.291 0.101	0.502 0.003	0.458 0.007	0.336 0.060	1	0.591 <0.001
HLP	CC *p*	0.413 0.017	−0.161 0.372	0.279 0.116	0.221 0.216	−0.125 0.496	0.591 <0.001	1

MAP, mean arterial pressure; HR, heart rate; SCPP, superficial capillary plexus perfusion; DCPP, deep capillary plexus perfusion; CCP, choriocapillaris perfusion; SLP, Sattler’s layer perfusion; HLP, Haller’s layer perfusion; CC, correlation coefficient; *p*, *p*-value

## Data Availability

The data of this study are available from the corresponding author, F.R., upon reasonable request.
